# MR liver imaging with Gd-EOB-DTPA: a delay time of 10 minutes is sufficient for lesion characterisation

**DOI:** 10.1007/s00330-012-2486-2

**Published:** 2012-05-30

**Authors:** C. S. van Kessel, W. B. Veldhuis, M. A. A. J. van den Bosch, M. S. van Leeuwen

**Affiliations:** 1 Department of Radiology, University Medical Centre Utrecht, Heidelberglaan 100, 3584CX Utrecht, The Netherlands; 2 Department of Surgery, University Medical Centre Utrecht, Utrecht, The Netherlands

**Keywords:** Gd-EOB-DTPA, Examination time, Dynamic contrast-enhanced MRI, Hepatobiliary imaging, Lesion characterisation

## Abstract

**Objectives:**

To assess whether, in patients with normal liver function, a hepatobiliary delay time of 10 min after Gd-EOB-DTPA injection is sufficient for lesion characterisation.

**Methods:**

In 42 consecutive patients with suspected focal liver lesions, dynamic MRI was performed after intravenous Gd-EOB-DTPA, followed by hepatobiliary phases at 5, 10 and 20 min. The following items were assessed at each hepatobiliary phase: parenchymal enhancement, contrast agent excretion in bile ducts, lesion enhancement characteristics (hypo-, iso-, or hyperintensity, rim enhancement, central non-enhancement), and contrast- and signal-to-noise ratios, separately for hypo- and hyperintense lesions.

**Results:**

Following enhancement, parenchymal signal intensity increased significantly up to 10 min (86.3%, *P* < 0.001), and subsequently stabilised (86.5% after 20 min, *P* = 0.223). Biliary contrast agent excretion was first observed in 2, 32 and 5 patients after 5, 10 and 20 min respectively. Hepatobiliary lesion enhancement characteristics observed after 5 min persisted during later hepatobiliary phases. CNR and SNR ratios increased significantly (*P* < 0.05) up to 10 min after enhancement without further increase at 20 min, in hypo- and hyperintense lesions.

**Conclusions:**

If lesion characterisation is the primary reason for performing MRI, a hepatobiliary delay time of 10 min after Gd-EOB-DTPA injection is sufficient in patients with normal liver function.

***Key Points*:**

• *Magnetic resonance imaging is now a first line of investigation of the liver.*

• *Optimal CNR and SNR are achieved 10 min after Gd-EOB-DTPA injection.*

• *Typical enhancement characteristics are observed early and do not change.*

• *Ten-minute hepatobiliary delay is sufficient for characterisation of focal liver lesions.*

## Introduction

Gadolinium ethoxybenzyl diethylenetriaminepentaacetic acid (Gd-EOB-DTPA) is a hepatocyte-specific contrast agent for magnetic resonance imaging (MRI) of the liver, introduced in 2004 (Bayer Schering Pharma, Berlin, Germany). The additional value of Gd-EOB-DTPA compared with other gadolinium-based contrast agents is the selective uptake of contrast material by hepatocytes with subsequent accumulation in the normal liver parenchyma, starting early after the dynamic phase and reaching a plateau after 10–20 min. Gd-EOB-DTPA differs from a similar hepatobiliary gadolinium agent gadobenate dimeglumine (Gd-BOPTA, Bracco Diagnostics, Princeton, NJ, USA) in two aspects: the percentage of contrast agent excretion in the bile (50 versus 5%) and timing of the hepatobiliary phase (10–20 versus 60–120 min) [1, 2]. Thus, Gd-EOB-DTPA is the first hepatobiliary MR contrast agent to allow acquisition of dynamic imaging in the arterial, portal and venous phases with a subsequent hepatobiliary phase in one examination [3, 4].

Promising results have been published with increased detection rates of especially small lesions after Gd-EOB-DTPA-enhanced imaging [5–8]. Recently, Lowenthal et al. observed higher detection rates for the hepatobiliary phases compared with the dynamic phases, emphasising the added value of the hepatobiliary phase [9].

Besides improved lesion detection, hepatobiliary images also allow for improved lesion characterisation, especially for differentiating hepatocellular lesions with functioning bile ducts, such as focal nodular hyperplasia (FNH), which exhibit iso- or hyperintensity during the hepatobiliary phase, from hepatocellular lesions without bile ducts, such as adenomas and most hepatocellular carcinomas, which lack enhancement in the hepatobiliary phase [10].

The duration of the time delay for the hepatobiliary phase is a matter of discussion and is dependent on the rate of contrast agent uptake by the hepatocytes [11]. The product brochure advises a 20-min delay for evaluation of lesion detection and characterisation, and most studies published so far obtained their data at 20 min post-contrast injection. One study suggested that an examination time of 10 min is sufficient for accurate lesion detection in up to 61% of patients; however, no recommendations were given on how to differentiate between patients who should undergo imaging at 10 or at 20 min [12]. Another study reported contrast agent excretion in the common bile duct in 100% of non-cirrhotic livers after 20 min versus 25% after 10 min [13].

Studies assessing the optimal timing for lesion characterisation are not yet available. Several studies assessed enhancement patterns during Gd-EOB-DTPA-enhanced MRI of different lesion types. However, most articles compared results of unenhanced MRI with those of contrast-enhanced MRI, without differentiation between dynamic and hepatobiliary phases, leaving the additional value of the hepatobiliary phases for lesion characterisation undefined [5–8]. Moreover, as these studies combined hepatobiliary phases at various time delays, it is not possible to identify the diagnostic value of the individual delay times.

Thus, the purpose of the current study was to assess whether, in patients with normal liver function, a hepatobiliary delay time of 10 min is sufficient for lesion characterisation. Therefore, parenchymal enhancement, contrast agent excretion in the bile ducts, lesion enhancement and lesion characterisation at 5, 10 and 20 min post-Gd-EOB-DTPA injection were compared.

## Materials and methods

### Patients

Between May 2007 and February 2010, 52 consecutive patients underwent a total of 57 MRI of the liver with Gd-EOB-DTPA for evaluation of known or suspected focal liver lesions. Patients with a history of parenchymal disease (i.e. cirrhosis, steatosis) or recent chemotherapy treatment were excluded as these factors may influence liver function and therefore Gd-EOB-DTPA uptake and excretion. This resulted in a final inclusion of 42 patients for this retrospective analysis: 36 patients with benign disease (focal nodular hyperplasia *n* = 18, adenoma *n* = 9, haemangioma *n* = 2, cysts *n* = 2, undefined benign lesions *n* = 3, no lesions *n* = 2), and 6 patients with malignant disease (colorectal liver metastases *n* = 3, hepatocellular carcinoma *n* = 1, intrahepatic cholangiocarcinoma *n* = 1, oesophageal carcinoma metastases *n* = 1). Diagnosis of focal liver lesions was histologically proven in 10 out of 42 patients (FNH *n* = 1, hepatocellular carcinoma *n* = 1, adenoma *n* = 5, colorectal liver metastases *n* = 3), either in surgically treated patients or by a biopsy specimen. In the remaining patients, no pathological confirmation was available, and these patients received follow-up with ultrasound, CT or MRI to confirm lesion diagnosis. Mean follow-up of these patients was 19 months (4–51 months), and one patient with an intrahepatic cholangiocarcinoma received only 2 months of follow-up before dying of cancer-related causes.

The study was approved by the ethics committee, and informed consent was waived as none of the patients was subjected to any additional test.

### MRI

MRI acquisitions of all patients were performed on a 1.5-Tesla MRI (Philips, Best, The Netherlands) using a SENSE Body Coil. The pre-contrast protocol consisted of the following sequences: breathhold T1-weighted turbo field echo (TFE) images (transverse and coronal), T1-weighted in- and out-of-phase images, free-breathing T1-weighted TFE images and free-breathing T2-weighted images (see Table 1). Administration of Gd-EOB-DTPA (25 μmol/kg) was carried out as a bolus with a rate of 2 mL/s through an intravenous cubital line, followed by a 25-mL saline chaser. After bolus injection, T1-weighted 3D-TFE was performed sequentially at 20, 60 and 180 s (dynamic phases) and at 5, 10 and 20 min post-contrast injection (hepatobiliary phases 5, 10 and 20 min respectively). Limited availability of MRI equipment precluded the acquisition of the 20-min series in 18/42 patients (43%). In these patients the entire MRI examination, including the 5- and 10-min series, was already assessed as being diagnostic. All images were acquired in the transverse plane with a section thickness of 4 mm, reconstructed every 2 mm. MRI data were stored in the Picture Archiving and Communication System (PACS) at the UMC Utrecht (Easy Vision Workstation, Philips Medical Systems, Best, The Netherlands), and further assessment of MRI acquisitions was performed on clinical PACS workstations.

**Table 1:** MRI pulse sequence protocol used during the study period: 1.5 T MRI, dedicated torso coil

Pulse sequence	Plane	TR	TE	Flip	FOV (mm)	Gap (mm)	Slice (mm)	Matrix
SURVEY insp	Axial	2.5	1.27	50	450	3.5	8	192 × 144
SURVEY exp	Axial	2.5	1.27	50	450	3.5	8	192 × 144
Refscan	Axial	8.0	0.57					56 × 40
T1 TFE bh, insp	Axial	8.5	4.2	10	450	0	10	256 × 128
T1 TFE bh, insp	Sagittal	8.5	4.2	10	450	0	10	256 × 128
T1 TFE bh, insp	Coronal	8.5	4.2	10	450	0	10	256 × 128
T1 TFE bh in + out of phase	Axial	181	2.3/4.6	80	375	1	7	224 × 134
T2 TSE RT	Axial	556	80	90	405	1	7	400 × 215
T1 FFE RT	Axial	10	4.6	15	405	1	7	256 × 126
T2 TSE SSH	Axial	8,000	800	90	300		40	320 × 256
EPI-DWI b = 0, 50 fb, RT	Axial	4,095	56	85	360	0	5	128 × 83
EPI-DWI b = 0, 500 fb, RT	Axial	4,095	56	85	360	0	5	128 × 83
THRIVE bh (pre-contrast; 25 and 60 s; 3, 5, 10 and 20 min)^a^	Axial	3.7	1.76	10	450	−2	4	176 × 124

*TR* Repetition time, *TE* echo time, *flip* flip angle, *FOV* field of view, *slice* slice thickness, *exp* expiratory, *insp* inspiratory, *TFE* turbo field echo, *TSE* turbo spin echo, *FFE* fast field echo, *EPI* echo planar imaging, *SSH* single shot, *RT* respiratory triggered, *bh* breath hold, *fb* free breathe, *THRIVE* T1-weighted high resolution isotropic volume examination

^a^After injection of Gd-EOB-DTPA-DTPA 0.25 μmol/kg at 2 ml/s

### Parenchymal contrast agent accumulation and contrast agent excretion in the bile

All MRI images were evaluated in order to obtain data on liver signal intensity, lesion signal intensity, muscle signal intensity and contrast agent excretion in the bile. The signal intensity measurements were performed on the pre-contrast acquisition and on each post-contrast phase by one investigator (C.v.K., research fellow). Signal intensity of the liver parenchyma was measured at three different points in the liver (in the right and left hemi-liver and at the midportion of the liver) in order to obtain an average signal intensity per sequence (mean SI_liver_). Signal intensity measures were obtained by means of circular, operator-defined regions of interest (ROI, approximately 100 mm^2^) avoiding any vessels, lesions or peripheral perfusion abnormalities. Measurements were performed at the same anatomical location on each sequence. The relative increase in mean SI_liver_ in time was measured in terms of percentage: (mean SI_liver_ _post_/mean SI_liver pre_) × 100%. The SI of the background noise was measured in an ROI that was placed just ventral to the liver and outside the body along the phase-encoding direction (ROI approximately 500 mm^2^).

Lesion signal intensity was measured once, and the ROI was set to contain as much of the lesion as possible, avoiding areas of necrosis or haemorrhage. Lesion-to-liver contrast-to-noise ratios (CNR) were calculated as follows: (SI_liver_ − SI_lesion_)/SD_noise_. Also, signal-to-noise ratios (SNR) were calculated as follows: SI_lesion_/SD_noise_. Analyses during the hepatobiliary phases were performed separately for hypointense and hyperintense lesions.

Furthermore, signal intensity of the left erector spinae muscle (SI_muscle_) was determined at each dynamic sequence, including the pre-contrast sequence. These intensity measures were used as the reference signal intensity for the liver. Relative increase in signal intensity of the erector spinae muscle was calculated [(SI_muscle post_/SI_muscle pre_) × 100%]. Liver-to-muscle contrast ratios (L-M ratios) were calculated as follows: L-M ratio = mean SI_liver_/SI_muscle_.

All hepatobiliary phases of each patient were assessed for contrast agent excretion in the bile ducts [13]. Contrast agent excretion was defined as contrast agent visible in the common bile duct.

### Enhancement characteristics

In order to ascertain the optimal delay time to assess the individual hepatobiliary enhancement patterns of the various liver lesions, 5-, 10- and 20-min hepatobiliary series were evaluated consecutively. Enhancement patterns were assessed by two observers in consensus (M.v.L. and C.v.K.). Enhancement patterns were categorised as isointense, hypointense or hyperintense relative to the normal liver parenchyma. In addition, heterogeneity, presence of rim enhancement or areas of linear or nodular non-enhancement were recorded.

### Statistical analysis

Data on signal intensities were displayed as mean ± standard error (percentages) or median with interquartile range (IQR; absolute values). Normal distribution of the data was tested using the Shapiro-Wilk’s test for normality and histograms. In parametric data, paired *t*-tests were used to test for significant differences in signal intensities of the liver, lesions, muscle, L-M ratios, CNRs and SNRs among 5, 10 and 20 min post-Gd-EOB-DTPA injection. In non-parametric data, the Wilcoxon signed ranks test was used. Statistical analyses were performed with commercially available software (SPSS version 15.0 for Windows, SPSS, Chicago, IL, USA). Statistical significance was set at a *P* value of less than 0.05. All analyses were performed in agreement with our in-house statistician.

## Results

### Patients and timing of delayed images

Three patients received two MRI during the study period as individual follow-up was clinically warranted. In those three patients, only the first MRI was included to prevent disproportionate contribution of these patients to the data. This resulted in a total of 42 MRI that were available for further analysis. Early hepatobiliary phase imaging at 5 and 10 min post-Gd-EOB-DTPA injection were obtained in all 42 patients, while 20-min imaging was obtained in 24/42 (57%) patients.

### Contrast agent excretion in the bile

Contrast agent excretion in the common bile duct was observed in 2/42 (4.8%) patients after 5 min and in 34/42 (80.9%) patients after 10 min. After 20 min, contrast agent excretion was observed in 21/24 patients, thereby resulting in a total of 39/42 (92.9%) patients showing contrast agent excretion at either 10 or 20 min post-contrast agent injection. Three patients did not show contrast agent excretion at any available sequence. The absence of contrast agent excretion could be explained in one patient by the presence of intrahepatic cholangiocarcinoma causing bile obstruction, in the second patient by the presence of multiple large adenomas affecting almost 70% of the liver parenchyma, whilst in the third patient no straightforward explanation for the delayed contrast agent excretion could be identified.

### Parenchymal enhancement

**Fig. 1:**
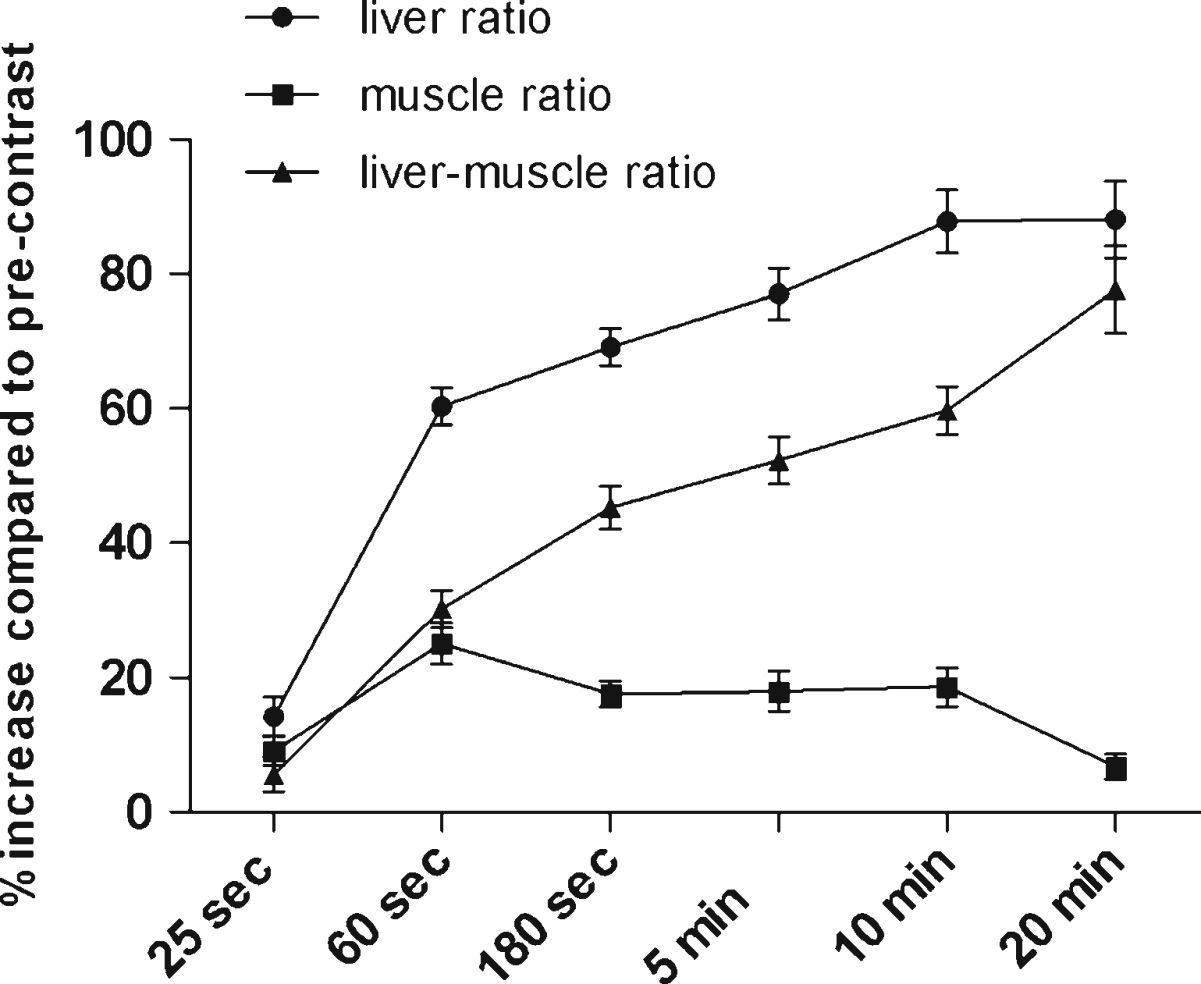
Mean (±SE) relative increase in signal intensities over time compared with the pre-contrast imaging. Results are displayed for the liver, the muscle and the liver-muscle ratios

Compared with pre-contrast SI_liver_, the median relative increase in SI_liver_ at 5, 10 and 20 min was 75.1% (IQR 18.6), 86.3% (IQR 28.8) and 86.5% (IQR 34.0) respectively. The difference between 5 and 10 min was significant (*P* < 0.001), whilst the difference between 10 and 20 min was not significant (*P* = 0.223). Signal intensity of the M. erector spinae was variable over time (see Fig. 1), with a significant decrease in signal intensity between 10 min (17.7%, IQR 12.4) and 20 min (4.1%, IQR 10.74; *P* < 0.001). This resulted in significant elevation of relative L-M ratios (%) at 5, 10 and 20 min (52.5 ± 3.7, 59.7 ± 3.6 and 77.65 ± 6.5% respectively) compared with pre-contrast sequences. The increases between 5 and 10 min and between 10 and 20 min were both significant (*P* < 0.001). However, the large difference between 10 and 20 min was mostly caused by the difference in relative signal intensity of the muscle at 10 min (17.7%) and 20 min (4.1%).

**Fig. 2:**
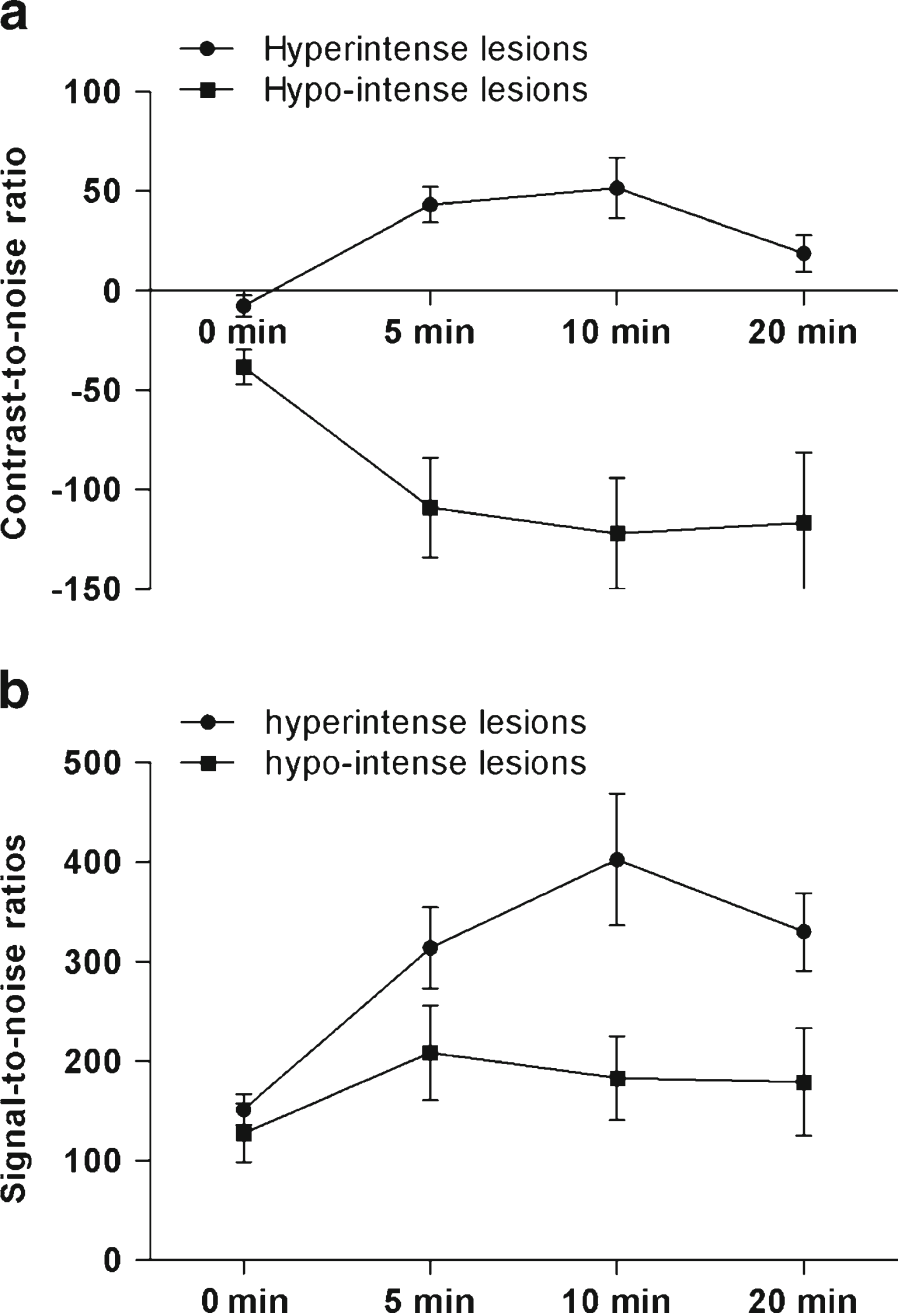
**a** Mean contrast-to-noise ratios with standard error of the mean at 5, 10 and 20 min compared with the pre-contrast images. **b** Mean signal-to-noise ratios with standard error of the mean at 5, 10 and 20 min compared with the pre-contrast images

The SNR and CNR were separately assessed for hyper- and hypointense lesions in the hepatobiliary phase respectively (Fig. 2a, b). For hyperintense lesions, CNRs at 5, 10 and 20 min were significantly increased compared with pre-contrast images (*P* < 0.05). However, CNRs were highest at 10 min, and a significant reduction was observed at 20 min (*P* = 0.039). SNRs showed a similar pattern: SNRs at 5, 10 and 20 min were significantly higher compared with pre-contrast images; however, SNRs were highest at 10 min while SNRs at 5 and 20 min were similar (*P* = 0.620). In hypointense lesions CNRs were significantly increased in the hepatobiliary phases compared with pre-contrast images (*P* < 0.05), but CNRs did not further improve between 5 and 10 min (*P* = 0.355), or between 10 and 20 min (*P* = 0.323; Fig. 2a, b). SNRs increased significantly compared with pre-contrast images (*P* < 0.05), however, compared with 5-min images, SNRs did not further improve at 10 min (*P* = 0.325) and 20 min (*P* = 0.937).

### Enhancement characteristics

**Fig. 3:**
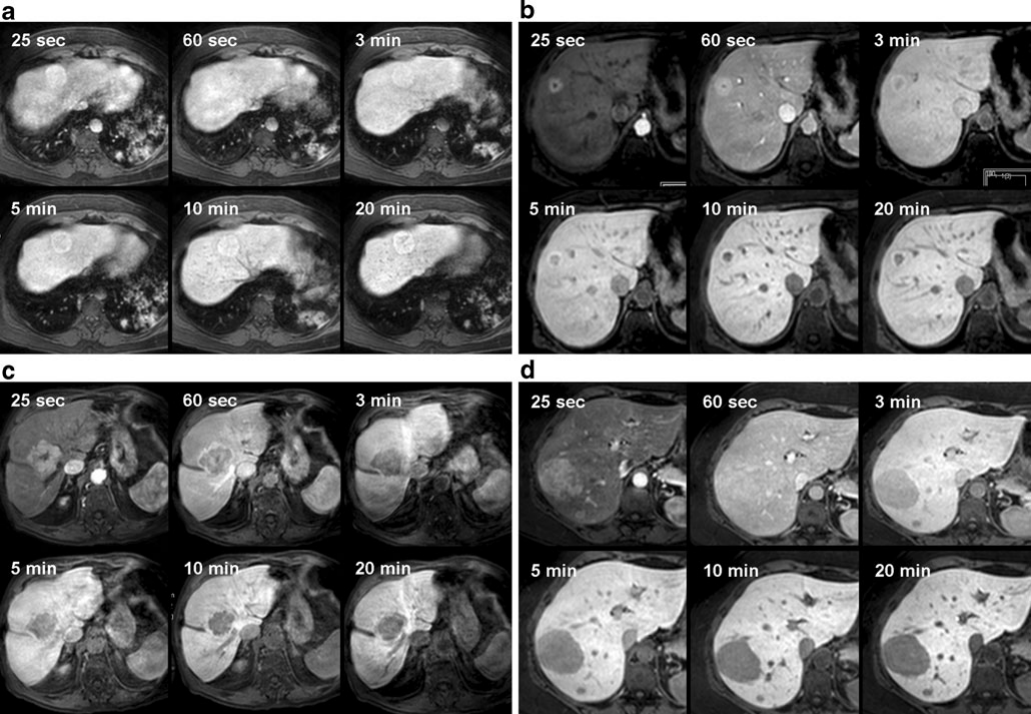
**a**-**d** Various enhancement patterns of solid hypervascular lesions during early dynamic phases and hepatobiliary phases (25 and 60 s; 3, 5, 10 and 20 min). **a** A 48-year-old man received an MRI with Gd-EOB-DTPA after he presented with an incidental lesion on ultrasound. The MRI shows a lesion in segments 4 and 8 of the liver, demonstrating the classic pattern of a focal nodular hyperplasia: hyperintense in the arterial phase, isointense in the portal phase, followed by a hyperintense appearance due to accumulation of contrast agent from 3 min onward, persisting into the later hepatobiliary phases. Furthermore, central linear non-enhancing structures in the hepatobiliary phases represent a central scar. The lesion remained stable in size during a 1.5-year follow-up. **b** A 59-year-old woman underwent abdominal CT during follow-up of a colorectal carcinoma. The lesion in segment 8 showed the following characteristics on MRI with Gd-EOB-DTPA: hyperintense on the arterial and portal phases with central hypointensity. After 3 min the central hypointense area enlarges, surrounded by a suggestion of contrast agent accumulation at the periphery of the lesion. During the later hepatobiliary phases the lesion centre becomes more hypointense compared with the liver parenchyma, and now unequivocal contrast agent accumulation at the periphery of the lesion is observed. These characteristics can occur in an atypical focal nodular hyperplasia (FNH) [10]. However, this patient had a history of malignancy and was therefore scheduled for surgery. Histopathology revealed FNH. **c** A 78-year-old man presented with the diagnosis of FNH based on a previous CT. A Gd-EOB-DTPA MRI was performed, showing a hypervascular lesion, with a central, non-enhancing cleft during the arterial phase, suggestive of a scar. During the portal phase, the central cleft remains, whilst the larger portion of the lesion is hypointense relative to surrounding parenchyma signifying wash-out. There is no contrast agent uptake during the subsequent hepatobiliary phases. As wash-out and non-accumulation in the late phases are atypical of FNH, a biopsy was performed. Histopathology revealed a well-differentiated hepatocellular carcinoma. **d** A 41-year-old woman presented with abdominal pain. Ultrasound revealed two large lesions in the right hemi-liver, and an MRI with Gd-EOB-DTPA was performed. The lesions are hyperintense on the arterial phase, iso-intense on the portal phase and homogeneously hypointense during all hepatobiliary phases. Thus, there is no contrast agent accumulation, and there are no signs of a central scar. This is an atypical finding, consistent with a hepatocellular adenoma or hepatocellular carcinoma. The patient underwent surgery, and histopathology revealed two hepatocellular adenomas

In 20 out of 42 patients multiple lesions were present, and in these patients enhancement characteristics of the various lesions were similar. Patients with multiple lesions presented either with adenomas (12/20), FNHs (6/20), or with colorectal liver metastases (2/20). In 17 patients lesions were identified with an overall hyperintense appearance during the hepatobiliary phases; in 5/17 (29%) patients the lesions were homogeneously hyperintense, in 4/17 (24%) patients the lesions showed heterogeneous hyperintensity with areas of hypointensity, in 7/17 (41%) patients the lesions were hyperintense with a central cleft-like hypointensity and 1 patient presented with a lesion with a narrow, peripheral hyperintense border, surrounding the hypointense central part of the lesion (shown in Fig. 3b). In all 17 patients, the observed enhancement characteristics at 5 min persisted during the later phases.

Another 18 patients presented with lesions with an hypointense appearance during the hepatobiliary phases. Most patients presented with homogeneously hypointense lesions (*n* = 17), and one patient presented with a lesion with a marked hypointense lesion centre. Again, these enhancement characteristics were observed after 5 min and persisted during later phases in all 18 patients.

Another patient presented with a solitary lesion that was only visible during the arterial phase and appearing isointense during all hepatobiliary phases. Four patients presented with lesions smaller than 5 mm that were too small to allow determination of the enhancement characteristics. In two patients no lesions could be detected.

In summary, the observed enhancement characteristics after 5 min, i.e. hypo-, iso- or hyperintense relative to the surrounding parenchyma, persisted after 10 and 20 min in all 36 patients harbouring lesions larger than 5 mm. Likewise, the presence of areas of non-enhancement or the presence of peripheral rim enhancement, once noted at the 5-min hepatobiliary phase, persisted during the later phases in all patients (Fig. 3).

## Discussion

The advantage of Gd-EOB-DTPA in combining early dynamic imaging with late, hepatobiliary phase imaging in one examination comes at the cost of the additional time required to obtain optimal lesion-to-liver contrast in the hepatobiliary phase.

In order to minimise examination duration the order of sequences can be adjusted. T2-weighted sequences can be acquired during the accumulation phase, however, once Gd-EOB-DTPA is excreted in the bile, the signal in the bile ducts will be lowered hampering assessment of the biliary tree during the hepatobiliary phase [8, 14, 15]. Nonetheless, if acquired immediately after the dynamic series, before biliary enhancement occurs, image quality is not impaired [14]. The T2-weighted imaging characteristics of lesions are not significantly influenced if acquired during the hepatobiliary phase [16]. Indeed, because of the lowering of the T2-weighted signal of the normal parenchyma in the hepatobiliary phase, lesion conspicuity may actually improve [16, 17]. Signal intensity on diffusion-weighted sequences is also not influenced in the accumulation phase, resulting in equivalent lesion conspicuity and apparent diffusion coefficients before and after Gd-EOB-DTPA injection [17–19]. These adjustments in the protocol can lead to a time reduction of several minutes.

In addition to rearranging the order of imaging sequences, the delay of the hepatobiliary phase should be limited to the time needed to achieve diagnostic image quality that allows adequate lesion characterisation and detection.

In this study, it is shown that in patients with normal functioning liver parenchyma, a delay time of 10 min after Gd-EOB-DTPA injection is sufficient to achieve adequate enhancement of the liver parenchyma. Contrast agent excretion in the bile ducts was visible in 81% of patients at 10 min suggesting contrast agent saturation of the parenchyma. The relative increase in parenchymal signal intensity between 5 and 10 min was statistically significant (75.1% at 5 min vs. 86.3% at 10 min respectively), whilst an additional 20-min series did not further increase the signal intensity.

Lesion characterisation and lesion detectability are parameters that are clinically more relevant than parenchymal enhancement. To our knowledge no studies have reported on the relative value of the various hepatobiliary delay times on lesion enhancement and characterisation. The current study shows that the individual hepatobiliary enhancement pattern, be it hypointense or hyperintense, depending on the type of lesion, is already observed after 5 min post-contrast injection. Once present, the observed individual enhancement pattern does not change thereafter and persists at the 10- and 20-min delay time. Also, in hypointense lesions, CNRs peaked at 10 min and then stabilised at 20 min, while SNRs showed a peak already at 5 min and then declined. In hyperintense lesions, both CNRs and SNRs were highest at 10 min and then decreased at 20 min. This again suggests that for characterisation of lesions an examination time of 10 min is sufficient.

**Fig. 4:**
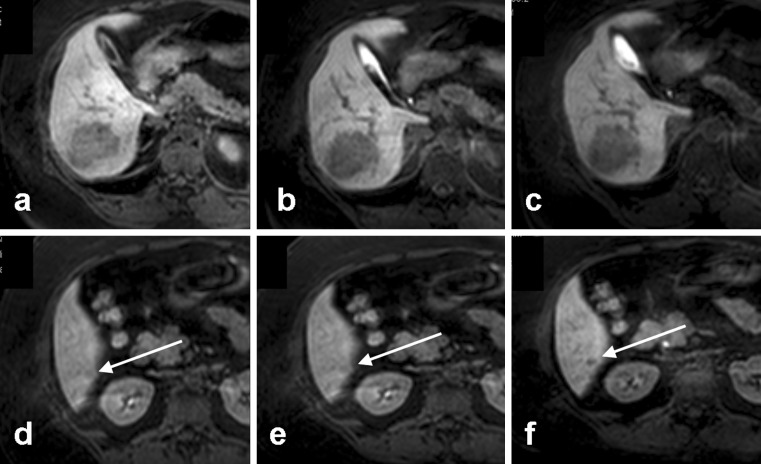
**a**-**f** In this patient, an additional lesion was detected at 20 min post-contrast injection. An adenoma in segment 6 is clearly visible at **a** 5, **b** 10 and **c** 20 min post-contrast injection. **f** An additional lesion was detected in segment 6 at 20 min post-contrast injection. In retrospective evaluation, with knowledge of the presence of this lesion at 20 min, a subtle hypointensity already reflects the presence of the lesion at **d** 5 and **e** 10 min

Regarding lesion detection, Motosugi et al. assessed whether it was possible to shorten the examination of the hepatobiliary phase to 10 min for imaging of (malignant) focal liver lesions, while maintaining adequate lesion detection [12]. They concluded that an examination time of 20 min could be omitted in 61% of cases, without loss of accuracy for lesion detection. A post-hoc analysis of the 24 patients in our study who received both 10- and 20-min acquisitions substantiated these results; the addition of 20 min to the combination of 5 and 10 min resulted in detection of six additional lesions in two patients (see Fig. 4.). Both of these patients were already diagnosed with multiple large adenomas, and the additional 10 min f led to the detection of six additional subcentimetre adenomas. No additional lesions were detected in the remaining 22 patients.

There are several limitations to this study. The patient selection is a skewed sample of the general population of patients with focal liver lesions, as there were 36 patients with benign lesions and only 6 patients with malignant lesions. However, this distribution reflects the main use of Gd-EOB-DTPA in our hospital, i.e., characterisation of solid liver lesions and especially the differentiation of FNH, which does not require treatment or follow-up, from other pathological conditions that do require further medical attention.

All patients underwent hepatobiliary phase acquisitions after 5 and 10 min. Because of the limited availability of the time slots for MRI, the hepatobiliary phase after 20 min was performed in only 24 out of 42 patients. In the remaining patients in whom the 20-min acquisition was not obtained, the combined 5- and 10-min series were already assessed as being diagnostic. However in the ideal situation all patients would have undergone 5-, 10- and 20-min imaging.

The ROIs of the erector spinae muscle were used as a reference to calculate liver-to-muscle ratios. Other articles assessing dynamic imaging (after Gd-EOB-DTPA) have used liver-to-spleen ratios to evaluate the degree of liver parenchymal enhancement, as the spleen is not affected by parenchymal diseases like the liver and contrast agent uptake is therefore not affected [12]. In this study, contrast enhancement of the spleen was fairly irregular, increased until 180 s and from that point gradually decreased (data not shown). Therefore, we used liver-to-muscle ratios with the erector spinae muscle as the reference, as reported in other articles [11, 20]. Nevertheless, our data showed a transient increase in contrast enhancement between 60 s and 10 min, which implies that the interstitial enhancement component of the muscle is delayed compared with that of the liver.

In conclusion, this study combined information on contrast agent uptake, contrast agent excretion and lesion enhancement characteristics. The results show that in patients without a history of chronic liver disease, steatosis or previous chemotherapy, a hepatobiliary delay time of 10 min after Gd-EOB-DTPA injection is sufficient if lesion characterisation is the main purpose of the study.
